# EVALUATION OF AN OFF-THE-SHELF, WEARABLE ACTIGRAPH SYSTEM FOR USE BY OLDER COUPLES

**DOI:** 10.1093/geroni/igad104.3311

**Published:** 2023-12-21

**Authors:** Cynthia Jacelon, Khadijat Adeleye, Raeann LaBlanc, Sara Mamo, Rebecca Spencer

**Affiliations:** University of Massachusetts Amherst, Amherst, Massachusetts, United States; University of Massachusetts Amherst, Amherst, Massachusetts, United States; University of Massachusetts Amherst, Amherst, Massachusetts, United States; University of Massachusetts Amherst, Amherst, Massachusetts, United States; University of Massachusetts Amherst, Amherst, Massachusetts, United States

## Abstract

There are many off-the-shelf wearable devices that provide the user with information regarding their physical activity and sleep patterns. Little is known about how easy it is for older individuals to use these devices. We tested a wrist-worn actigraph on couples both of whom were at least 70 years old. The aim was to evaluate the usability of the device, and to share data within the couples. Mixed methods were used to collect data. Over the three-week trial, 14 couples, nine of which had one member with mild cognitive impairment, used the system. Each person wore a Garmin Vivofit 4 continuously and used the Garmin application installed on an iPad mini to compare and review results over time. At the end of weeks one and three, participants completed the positively scored System Usability Scale (SUS), and each week met with the researcher to discuss the data and system usability. All couples were able to work together to use the system after being trained. Scores on the SUS indicated the system was moderately easy to use. Qualitative findings were that most participants (88%) liked being able to wear the device continuously, without breaks for charging. They compared information about their activity and sleep patterns within the dyad. There were several age-specific usability challenges, but use of off-the-shelf actigraphs by older individuals holds promise if age-related physical and cognitive changes are considered during selection of the device.

